# Early Neonatal Epilepsy Caused by Homozygous Mutation in the SLC13A5 Gene: A Case Report From India

**DOI:** 10.7759/cureus.74405

**Published:** 2024-11-25

**Authors:** Khalid M Saifullah, Snigdha Samanta, Ankit Ranjan, Rajesh Kumar

**Affiliations:** 1 Pediatrics and Neonatology, Rani Hospital and Research Centre, Ranchi, IND; 2 Neonatology, Postgraduate Institute of Medical Education and Research, Chandigarh, IND

**Keywords:** anti-convulsants, epileptic encephalopathy, genetic epilepsy, neonatal epilepsy, slc13a5 gene, whole exome sequencing

## Abstract

Early neonatal seizures have myriad causes and variable prognoses. While acute symptomatic seizures are the most common events, a significant number of cases have a genetic background for such seizures, and a timely diagnosis can help in appropriate management and prognostication. We present a case of a neonate referred to our center with multi-focal clonic seizure starting from the first day of life. Routine metabolic, radiological, and electrographic studies failed to unravel the cause, necessitating whole exome sequencing (WES), which revealed a homozygous deletion of the SLC13A5 gene on chromosome 17. The patient's parents' Sanger sequencing confirmed heterozygous mutation at the same loci, consistent with an autosomal recessive inheritance. This is perhaps among the few case reports of neonatal epilepsy associated with such mutation reported from India; however, the literature on this topic is growing worldwide.

## Introduction

Neonatal-onset seizures are mainly caused by acute symptomatic events like hypoxic-ischaemic encephalopathy, metabolic derangements, vascular insults, and infection. However, about 15-20% of neonatal seizures lack such acute causes, and investigations like next-generation sequencing often reveal a genetic underpinning [1]. Developmental epileptic encephalopathy (DEE) is a term coined to denote the devastating effect of epilepsy on neurodevelopmental outcomes at such a vulnerable age [2]. Most developmental epilepsy syndromes are caused by well-known mutations in genes like KCNQ2, SCN2A, KCNT1, KCNA2, STXBP1, and ALDH7A1 [3]. However, over the last decade, several large case series have identified other mutations that cause early neonatal epilepsy, which has an adverse impact on neurodevelopment. We report one such mutation in the SLC13A5 gene in a patient who presented with a seizure on day one of life.

The SLC13A5 gene codes a protein called sodium-coupled citrate transporter (NaCT), which is highly expressed in the brain, teeth, liver, and testes [4]. Citrate is an important intermediary in energy pathways of tricyclic carboxylic acid and fatty acid and its decreased level in neurons due to SLC13A5 loss of function results in impaired synthesis of GABA and, possibly, altered function of NMDA receptors, thereby increasing the tendency for seizures [5]. How SLC13A5 mutation leads to neurodevelopmental impairment in the absence of ongoing seizure at a later age is not fully understood [6]. Also, the loss of function of this gene in animal experimental models (mice and flies) has not resulted in neurological impairment; rather, it resulted in increased longevity [7]. The cardinal feature of SLC13A5 mutation is epilepsy with onset in the first week of life and impaired development of neurocognition later on [8].

The most commonly described epileptic phenomena are focal clonic, tonic-clonic, and status. Interestingly, unlike other epileptic encephalopathy presenting in newborns, gross EEG abnormalities like burst suppression are not seen and interictal EEG is often normal in these patients [6]. After infancy, epileptic attacks often wane and psychomotor impairment develops in the majority of cases. Hypotonia, choreoathetosis, absence of pyramidal signs, and profoundly poor communication skills are the other characteristic features of this mutation [8]. Dental anomalies like hypoplasia and hypodontia are also commonly associated with this disorder [9]. Various drugs have been tried to control these seizures but there is no drug of choice currently. Gamma-aminobutyric acid (GABA) mimetics like valproate and sodium-channel blocker carbamazepine have been tried in different case series with favorable results, while carbonic anhydrase inhibitor acetazolamide has demonstrated mixed results [10].

## Case presentation

The patient was a female infant born to a 32-year-old primigravida mother after a non-consanguineous marriage. The three-generation pedigree analysis had shown no epilepsy or any other neurological disorders in the family. Pregnancy had been mostly uncomplicated, except at term, when there had been meconium-stained liquor due to which a C-section had been performed. The baby had made a smooth perinatal transition and roomed in with the mother. At six hours of life, the parents had noticed brief jerky movements in upper limbs, which had not been considered significant. The jerky movements had continued during subsequent days, and she had been taken to a local hospital.

**Figure 1:**
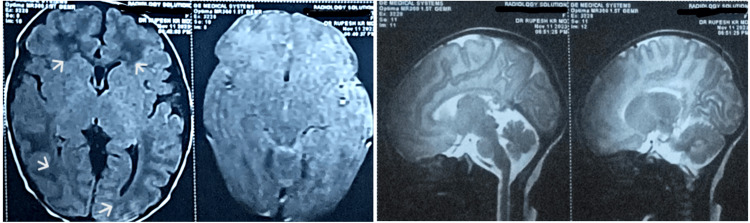
Brain MRI showing mild hypoxic changes in bilateral fronto-parietal and occipito-parietal regions White arrows show the hypodense areas MRI: magnetic resonance imaging

The initial metabolic workup and sepsis screening had been negative and the patient had been started on injection levetiracetam and phenobarbitone. Frequent multi-focal seizures lasting 20-30 seconds had persisted in both upper and lower limbs in a random fashion, in both sleep and awake state, prompting a referral to our hospital. A full sepsis screen including spinal fluid examination was normal. MRI brain showed mild hypoxic changes in the bilateral frontoparietal area (Figure 1).

**Figure 2:**
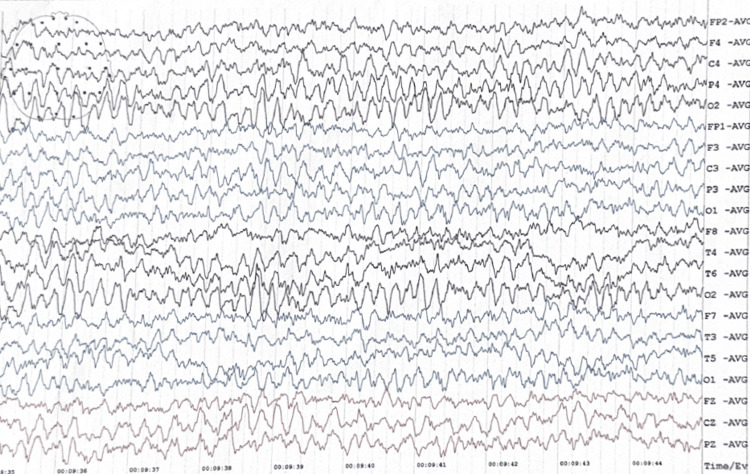
EEG performed after discharge during the interictal phase showing abnormal theta waves in right occipital lobe Overall, the background activity is normal EEG: electroencephalogram

Since the baby had good muscle tone, normal reflexes, and sucking well with intact sensorium right from birth, the cause of seizure being hypoxic-ischemic encephalopathy was considered unlikely. Continuous augmented EEG monitoring showed a good correlation with clinical seizure with bilateral hemispheric discharges in frontal and parietal areas. Background voltage always remained normal with no burst suppression. Interictal EEG findings were unremarkable except for abnormal theta activity in the right occipital lobe, the significance of which could not be explained (Figure 2).

Given uncontrolled seizure activity, multiple drugs were tried after reaching the maximum recommended doses of phenobarbitone and levetiracetam, including valproate, fosphenytoin, topiramate, and oxcarbazepine. As per our unit policy, we gave pyridoxine phosphate in tablet form. At 35 days of life, the baby developed nosocomial sepsis and meningitis (Klebsiella pneumonia) and received a full three-week course of IV antibiotics. After two months of life, seizure frequency started decreasing and the baby was weaned from valproate, fosphenytoin, and oxcarbazepine and discharged home on syrup phenobarbitone, syrup levetiracetam, and tablet topiramate.

Due to a strong suspicion of genetic/metabolic cause for epilepsy, a detailed metabolic workup (TMS and GCMS) and whole exome sequencing (WES) with an extended mitochondrial panel was requested after obtaining parental consent. The metabolic screen result was negative. The whole exome sequence report is mentioned below. At four months, the patient had a single episode of epileptic spasm in the upper limbs lasting a few seconds, which required no additional treatment. The last follow-up at five months of age showed no further seizure activity, and the patient is continuing with the three anticonvulsants prescribed at discharge. Her weight and length are below the third centile of the WHO growth chart, while her head circumference is between the 3rd and 15th centile. She has mild truncal hypotonia and she is responding well socially. She has been referred for early rehabilitation and neural stimulation and her parents have been advised to attend long-term follow-ups.

Genetic test results

WES results showed a missense homozygous mutation in the SLC13A5 (NM_177550.5) gene on Exon 5 of chromosome 17 (Table 1).

**Table 1:** Whole exome sequencing report of the case The findings showed homozygous deletion of the SLC13A5 gene on exon 5 of chromosome 17

Gene and transcript	Variant	Location	Zygosity	In silico parameters	Disorder (OMIM)	Inheritance	Variant classification
SLC13A5 (NM_177550.5)	c.655G>A (p.Gly219Arg)	Exon 5	Homozygous	CADD: 25.5, SIFT: deleterious MT: damaging	Developmental and epileptic encephalopathy 25, with amelogenesis imperfecta (615905)	Autosomal recessive	Pathogenic

This mutation resulted in amino acid substitution p.Gly219Arg. The observed variant is novel in genomes and the severity of the impact of this variant on the protein is high based on the effect of the protein and Rare Exome Variant Ensemble Learner (REVEL) score. This variant is classified as pathogenic by ClinVar and observed in patients with epileptic encephalopathy; hence, it was classified as pathogenic according to the American College of Medical Genetics (ACMG) guidelines.

WES also identified a heterozygous missense mutation in the SLC2A1 gene, a mutation also associated with epileptic encephalopathy at an early age, mostly as absence epilepsy before four years of age. However, based on the REVEL score, this variant was classified as Unknown Significance as per ACMG guidelines.

**Figure 3:**
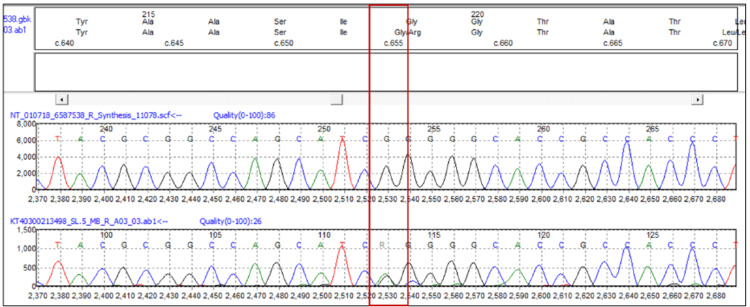
Sanger sequencing data (electropherogram) for the provided sample showing nucleotide change at chr17:c.655G>A (p.Gly219Arg) in the SLC13A5 gene in the father

**Figure 4:**
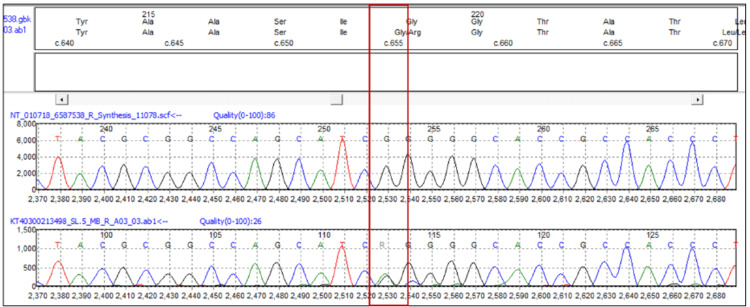
Sanger sequencing data (electropherogram) for the provided sample showing nucleotide change at chr17:c.655G>A (p.Gly219Arg) in the SLC13A5 gene in the mother

Parental testing was requested to identify the significance of these variants. Both parents were found to have a heterozygous deletion of SLC13A5 genes at the same loci (Table 2; Figures 3-4).

**Table 2:** Sanger sequencing of both parents confirming heterozygous deletion of SLC13A5 genes on same loci of chromosome 17

Sample	Gene name	Variant tested	Variant status	Inheritance
Father	SLC13A5	chr17:c.655G>A (p.Gly219Arg)	Present (heterozygous)	Autosomal recessive
Mother			Present (heterozygous)	

## Discussion

This is perhaps among the first few case reports of early neonatal epilepsy due to SLC13A5 mutation from India. Recently, Mondkar et al. [11] reported a case of SOFT (short stature-onychodysplasia-facial dysmorphism-hypotrichosis) syndrome with Kohlschütter-Tönz syndrome (eponym of SLC13A5 deficiency disorder). Their case was evaluated for short stature and the child presented at 2.2 years of age with dysmorphic facial features. There was a history of neonatal-onset seizure and the child was treated with levetiracetam and lacosamide.

In their original description of SLC13A5 mutation, Thevenon et al. [8] discussed seven cases who had severe epilepsy with neonatal onset and profound developmental delay with no facial dysmorphism. The cases had both compound heterozygous and homozygous mutations in the SLC13A5 gene. Unlike our case, which had predominant focal clonic seizures, their cohort had subclinical or polymorphic seizures. Quite similar to our case, Hardies et al. [9] reported focal clonic seizure on day one of life in their series of eight patients. They identified seven different autosomal recessive inherited variants in SLC13A5 in four independent families of European origin. Interictal EEG was normal in one of their cases. They successfully tried the ketogenic diet in three cases. Interestingly, all their patients had teeth hypoplasia, which has to be considered in our case once she grows older. Children in both case series developed various psychomotor problems at later ages while their seizure frequency decreased.

In a large case series, Yang et al. [4] reported 23 cases ranging in age from three months to 29 years. In all of them, seizures began in neonatal age and only a minority of them achieved seizure freedom. The EEG showed a well-preserved background and occasional slowing or normal records. Most of these patients had severe cognitive delays and profound motor and cognitive skills. However, unlike our case, the most common type of seizure reported was generalized tonic-clonic, atypical absence, and myoclonic. Only one case had spasms like in our case. Parents reported GABAa receptor agonists to be the best anti-convulsant medicine to control the seizure. Based on these case series and our case, a developmental epileptic encephalopathy seems a less likely diagnosis as the EEG background was mostly normal and the children continued to deteriorate in psychomotor development despite good epilepsy control. We believe there might be some other unknown mechanism behind such a phenomenon.

## Conclusions

While the SLC13A5 gene mutation can cause severe uncontrolled neonatal seizure, the unique characteristic of ongoing developmental encephalopathy in the absence of persistent epileptic activity at a later age is hitherto unexplained and needs further research. Genetic epilepsy should be considered early in differential diagnostic algorithms in cases where recurrent seizures cannot be explained by common acute etiology and routine investigations.
